# Combinatorial Roles of Heparan Sulfate Proteoglycans and Heparan Sulfates in *Caenorhabditis elegans* Neural Development

**DOI:** 10.1371/journal.pone.0102919

**Published:** 2014-07-23

**Authors:** Tarja K. Kinnunen

**Affiliations:** Institute of Integrative Biology, University of Liverpool, Liverpool, United Kingdom, and Department of Biology, University of Huddersfield, Huddersfield, United Kingdom; University of Patras, Greece

## Abstract

Heparan sulfate proteoglycans (HSPGs) play critical roles in the development and adult physiology of all metazoan organisms. Most of the known molecular interactions of HSPGs are attributed to the structurally highly complex heparan sulfate (HS) glycans. However, whether a specific HSPG (such as syndecan) contains HS modifications that differ from another HSPG (such as glypican) has remained largely unresolved. Here, a neural model in *C. elegans* is used to demonstrate for the first time the relationship between specific HSPGs and HS modifications in a defined biological process in vivo. HSPGs are critical for the migration of hermaphrodite specific neurons (HSNs) as genetic elimination of multiple HSPGs leads to 80% defect of HSN migration. The effects of genetic elimination of HSPGs are additive, suggesting that multiple HSPGs, present in the migrating neuron and in the matrix, act in parallel to support neuron migration. Genetic analyses suggest that *syndecan*/*sdn-1* and *HS 6-O-sulfotransferase*, *hst-6*, function in a linear signaling pathway and *glypican*/*lon-2* and *HS 2-O-sulfotransferase*, *hst-2*, function together in a pathway that is parallel to *sdn-1* and *hst-6*. These results suggest core protein specific HS modifications that are critical for HSN migration. In *C. elegans*, the core protein specificity of distinct HS modifications may be in part regulated at the level of tissue specific expression of genes encoding for HSPGs and HS modifying enzymes. Genetic analysis reveals that there is a delicate balance of HS modifications and eliminating one HS modifying enzyme in a compromised genetic background leads to significant changes in the overall phenotype. These findings are of importance with the view of HS as a critical regulator of cell signaling in normal development and disease.

## Introduction

Heparan sulfate (HS) and HS proteoglycans (HSPGs) critically regulate a range of biological processes in metazoan organisms from early events in gastrulation to adult physiology, disease and host response to pathogens (for reviews see [1], [2], [3]. The involvement of HS/HSPGs in such a broad range of biological activities is due to their ability to interact with a vast repertoire of proteins such as growth factors, morphogens, enzymes, extracellular matrix and plasma proteins. The molecular interactions of HSPGs occur predominantly via the negatively charged HS glycans attached to the proteoglycan protein core. The biosynthesis of HS occurs in the Golgi complex, where a set of polymerases first synthesize the polysaccharide backbone consisting of *N*-acetylglucosamine (GlcNAc) and glucuronic acid (GluA) residues. The linear HS backbone undergoes a complex pattern of modifications at selective positions. The first modification step is *N*-deacetylation and subsequent *N*-sulfation of some glucosamine units. Subsequent epimerization of some GluA units to iduronic acid (IdoA) and sulfation at C2 hydroxyl group of GluA/IdoA and sulfation at C6 or C3 of the glucosamine are actions of a glucuronyl C5-epimerase and heparan 2-, 6- and 3-O-sulfotransferases, respectively. These modifications generally occur in the *N*-sulfated regions creating domains of high sulfate density (NS-domains), intermediate sulfate density (NS/NA-domains) and low sulfate density (NA-domains). HS biosynthesis is not template-driven and enzymatic reactions do not modify every sugar unit, resulting in huge structural complexity in one of the most heterogeneous molecules in biology.

In vertebrates, most of the HS biosynthetic enzymes are encoded by members of multigene families. Multiple isoenzymes for HS polymerases (EXT/EXTL) [4], [5], [6], four *N*-deacetylase/*N*-sulfotransferases (NDSTs; [7], three glucosaminyl 6-O-sulfotransferases (HS6STs; [8] and seven isoforms of glucosaminyl 3-O-sulfotransferases (HS3STs; [9] have been identified to date. The specific isoenzymes possess differential substrate specificities. Only a single glucuronyl C5-epimerase [10] and uronyl 2-O-sulfotransferase (HS2ST; [11] have been identified to date.

Biochemical analyses have revealed developmental and disease related changes to HS composition. Gene knock-out studies have identified key signaling pathways and developmental processes that require the activities of specific HS biosynthetic enzymes. For example, genetic elimination of mouse *Hs2st* leads to complete lack of kidneys and neonatal lethality [11]. Targeted disruption of C5-epimerase in mice leads to renal agensis, lung defects and skeletal malformation [12]. Targeted disruption of HS biosynthetic enzymes is expected to affect all different HSPGs expressed in the targeted tissue or developmental stage. Similarly, most biochemical analyses to structural changes in HS have used whole animal or tissue samples containing a pool of HSPGs. There is limited data on the relationship between HS structures and specific core proteins and whether the core protein influences structural specificity of the HS. Mouse syndecan and glypican HSPGs ectopically expressed in immortalized cell lines are decorated with HS of similar structural composition [13] suggesting that at least *in vitro*, the core protein context is not critical for HS structure determination. Syndecan and glypican from rat embryonic fibroblasts carry HS chains of different molecular mass, but similar charge and structural composition, yet display differential binding to fibronectin, suggesting that the core protein may determine ligand specificity [14]. However, syndecan-3 purified from perinatal rat forebrain is decorated with HS that contains a higher degree of *N*-sulfation and IdoA units as compared to average tissue HS [15] suggesting that the structural regulation of specific HS is more complex *in vivo*.

The *C. elegans* hermaphrodite specific neurons (HSNs) are a pair of serotonergic neurons that innervate the vulval muscles and are necessary and sufficient to initiate egg laying [16]. The HSNs are born in the tail of the embryo and during embryonic development the HSNs migrate to mid body, to the immediate proximity of the developing vulva. Defects in HSN development often result in egg-laying defects, which can be readily observed as accumulation of embryos inside the mother.

This study sought to address the relation between a specific HSPG core protein and distinct HS motifs using a neural development model in *C. elegans in vivo*. *C. elegans* has in most cases a single orthologue of vertebrate genes encoding for HSPG core proteins and HS biosynthetic enzymes facilitating analysis without genetic redundancy [17], [18], [19], [20], [21], [22], [23], [24]. Developmental genetic approach was used to assess the relation between specific HS structures on different HSPG core proteins for the migration of the HSNs. The HSNs undergo a significant developmental migration in relation to the *C. elegans* body plan, and were thus chosen as a model to assess HSPGs and HS in neural development. Multiple HSPG dependent neuron guidance pathways are required for HSN migration in parallel. These parallel pathways rely on different HS modifications. Genetic analysis also suggests an interdependence of HS biosynthetic enzymes during HS biosynthesis.

## Materials and Methods

*Strains- C. elegans* strains were maintained at 20°C essentially as described [25] unless otherwise stated. Wild type strain used in this study is N2 var. Bristol. The following previously described mutations were used: LGI: *cle-1* (*cg120*) [22]; LGIII: *hse-5 (tm472)* [21], *rib-2* (*tm710*) [23]; LGIV: *rib-1* (*ok556*) [24], *hst-1* (*ok1068*); LGX: *lon-2 (e678)* [18], *hst-6 (ok273)* [17], *sdn-1(zh20)* [26], *sdn-1(ok449)* [20], *hst-2(ok595)* [19], [21], [26], *gpn-1 (ok377*) [27]. *tm734* and *tm3006* are deletion alleles of *hst-3.1* and *hst-3.2,* respectively, generated by Shohei Mitani and the Japanese National Bioresource Project. *tm734* is a 448 bp deletion and 4 bp insertion eliminating exons 2 and 3 and parts of exon 4 and *tm3006* is a 230 bp deletion removing parts of exons two and three. Both mutant alleles were backcrossed at least three times prior to analysis. Both mutants are homozygous viable and fertile and display no overt phenotypic defects as single mutants. The following transgenes were used; *zdIs13* [P*tph-1*::GFP] [28], *mgIs71* [P*tph-1*::GFP;*rol-6(d)*] [29]. Double and triple mutants were generated using standard genetic methods.

### Reporter constructs

Standard molecular biological techniques were used. The *sdn-1::gfp* transcriptional reporter contains 1.5 kb of sequence upstream from predicted ATG site cloned into SphI/SmaI sites of pPD95.75. The *hst-3.1::gfp* and *hst-3.2::gfp* reporters contain a 2 kb of sequence upstream from corresponding predicted ATG site cloned into SphI/XbaI and SphI/BamHI restriction sites of pPD95.75, respectively. DNA constructs were injected at 30 µg/ml. p*ttx-3::*mCherry or p*tph-1*::mCherry were used as injection markers at 50 µg/ml and pBluescript was used as a filler DNA.

### Neuron scoring

HSN neuron migration was scored using cell specific fluorescent reporters that are expressed in the HSNs. Each *C. elegans* has two HSNs but sometimes it was not possible to see both HSNs due to the position of the animal. Neuron scores are thus presented as number of total neurons scored, rather than number of animals scored, unless otherwise stated. Statistical significance was tested using either Student’s t-test or Fisher’s test.

### Microscopy

Microscopy of living *C. elegans* was performed by mounting the animals on a 4% agarose pad in a drop of M9 buffer containing 30 mM NaN_3_ as an anesthetic. HSN migration was scored under fluorescent microscopy using GFP expression in all serotonergic neurons including the HSNs. Compound fluorescent images were acquired using Zeiss AxioCam MRm camera mounted on Zeiss Axioskop2 microscope equipped with x20, x40 and x63 optics. Images were captured using Axiovision. Confocal images were acquired using Leica AOBS SP2 system and images were captured using Leica software. Microscopy images were further cropped and scaled using Adobe Photoshop CS3 (Adobe Systems, San Jose, CA).

## Results

### Parallel HSPG dependent pathways are required for HSN migration

**Figure 1:**
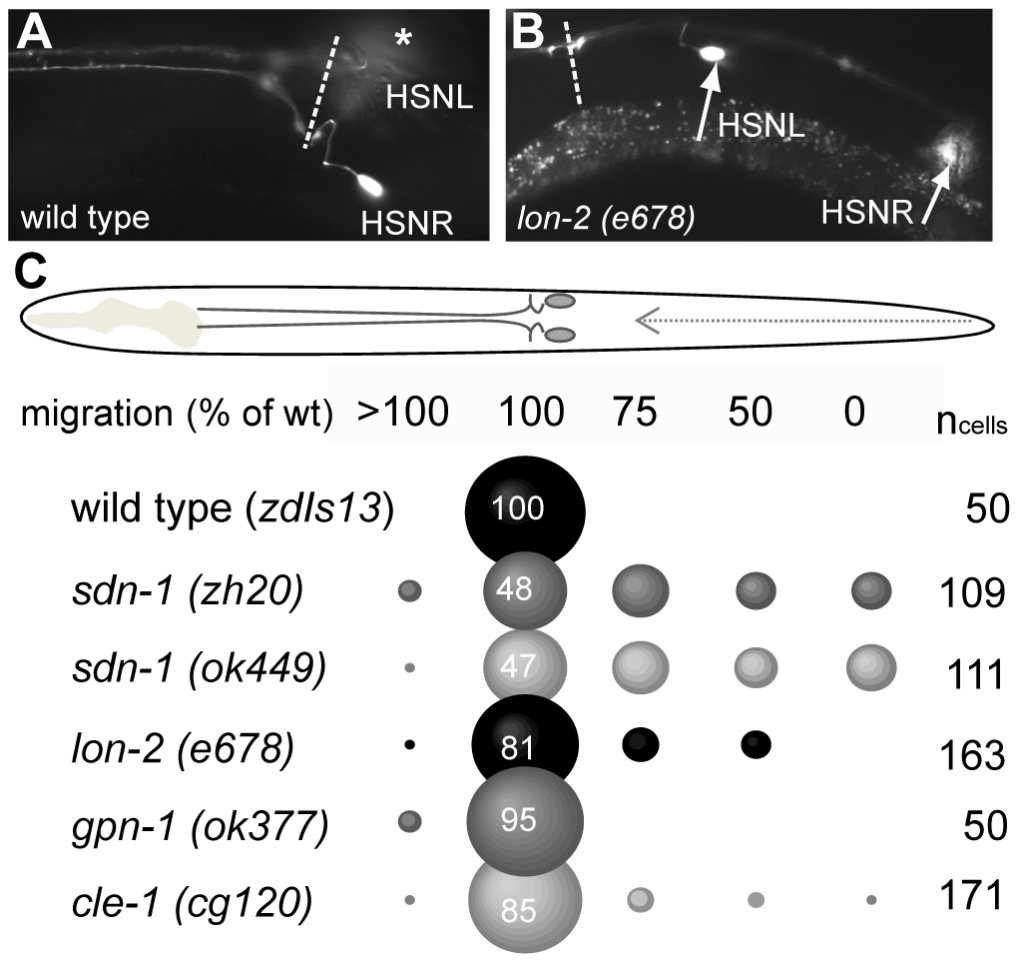
Multiple HSPGs are required for migration of hermaphrodite specific neurons (HSNs). (A) The HSNs visualized using *ptph-1*::GFP expression (*zdIs13*) in wild type background, ventral view. Left and right HSNs are labeled. Left HSN (HSNL) is out of focal plane and is marked with an asterisk. The position of the vulva is marked with a dashed line. The HSN cell bodies, which are positioned posterior to the vulval opening, send axons along the left and right ventral nerve cord to the nerve ring in the pharynx. A branch of these axons innervates the vulval muscles. (B) HSN migration defect in glypican/*lon-2* (*e678*) mutant, lateral view. Arrows point to the HSN cell bodies which are both posteriorly misplaced. The position of the vulva is marked with a dashed line. Anterior is to left and posterior is to right in both (A) and (B). (C) Schematic drawing shows ventral view of the HSNs within the *C. elegans* body plan. The HSNs are born in the tail of the embryo and as the embryo begins to elongate the HSNs migrate through the posterior part of the animal (dashed arrow). The position of HSNs in HSPG mutant background was scored as percentage of neuron migration where 0% is no migration, 100% is wild type neuron position and >100% is over-migration (anterior to vulva). The position of the bubble represents the position of the neurons. The size of the bubble indicates percentage of HSN cell bodies that have migrated to each position; numbers indicate percentage/size of the bubble. All strains carry *zdIs13*. n_cells_; number of neurons analyzed for each strain.

Unlike mammals which have four syndecan and six glypican genes, the genetic redundancy in *C. elegans* HSPGs more restricted. *C. elegans* has two orthologues of glypicans, *gpn-1* [27] and *lon-2* [18] and a single orthologue of syndecan, *sdn-1* [20], collagen XVIII, *cle-1* [22] and perlecan, *unc-52* [30]. Mutants in syndecan/*sdn-1* display the strongest HSN migration defect. A null allele of *sdn-1, zh20,* [26] and an in-frame deletion allele *ok449* display 52% and 53% HSN migration defects, respectively (Fig. 1). Notably, 12% (*zh20*; 12 out of 111 neurons) and 17% (*ok449*; 18 out of 109) of all the HSNs failed to migrate at all in *sdn-1* mutants (Fig. 1). In glypican/*lon-2 (e678)* mutants all the HSNs undergo partial migration but 19% (31 out of 163) fail to reach wild type position (Figure 1), whereas single mutants in the other glypican, *gpn-1* (*ok377*), have wild type HSN migration. Collagen XVIII/*cle-1* (*cg120*) mutants displayed 15% defect in HSN migration (Fig. 1).

**Figure 2:**
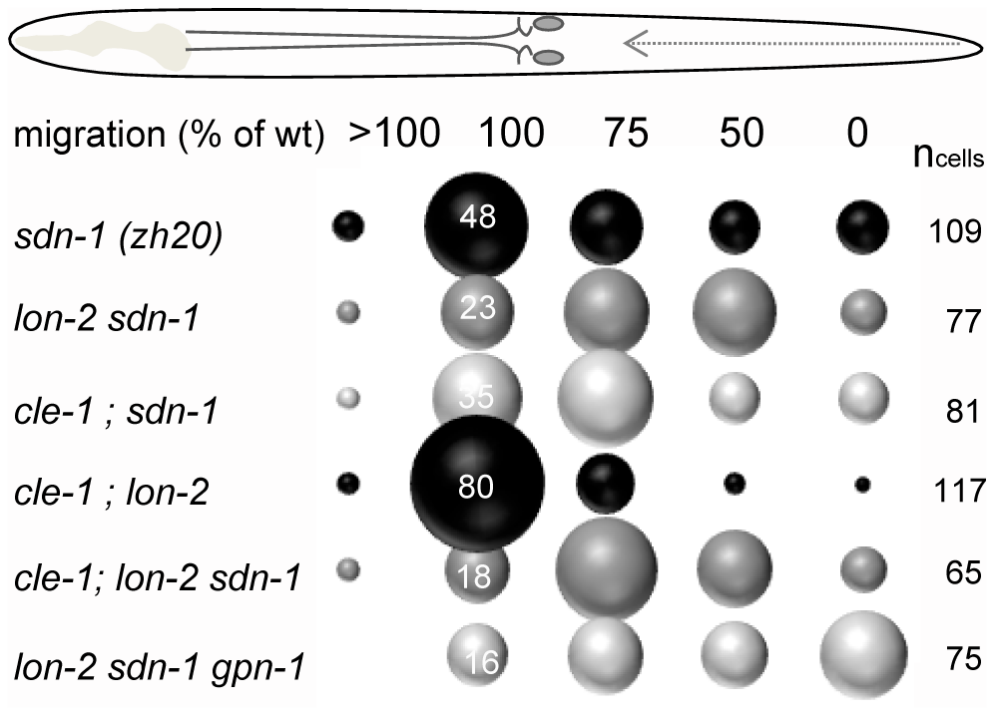
Multiple HSPGs are required in parallel guidance pathways for neural migration. Double mutants in HSPG core proteins display more severe neuron migration defects than single mutants and the defects are enhanced in triple mutants. Note that although mutations in *gpn-1 (ok377)* alone do not result in defects in HSN migration, removing *gpn-1* from *sdn-1 lon-2* mutant results in significantly more neurons failing to migrate at all. n_cells_; number of HSNs analyzed for each strain.

To test whether the different HSPGs have redundant functions or whether each HSPG has its own role in HSN migration, HSPG mutants were analyzed in combinations. Mutations in *lon-2* (*e678*) and *cle-1* (*cg120*) enhanced HSN migration defects of *sdn-1* (*zh20*) from 52% (n = 111) to 77% (n = 77; *P*<0.001) and 65% (n = 81) respectively (Fig. 2). Similar enhancement of HSN migration defect was observed in combination with the *sdn-1 ok449* allele. The neuron migration defect was further enhanced to 82% (47 out of 62 cells) in *cle-1 lon-2 sdn-1* triple mutants (Fig. 2), suggesting that CLE-1/collagen XVIII, LON-2/glypican and SDN-1/syndecan play independent roles in parallel neuron migration pathways. Interestingly however, double mutants in *lon-2* (*e678*) and *cle-1* (*cg120*) did not display significantly enhanced HSN migration defect as compared to either single mutant alone (20% HSN migration defect in *cle-1 lon-2* double mutant; n = 117). Mutations in *gpn-1*/glypican did not significantly enhance the HSN migration defect of *sdn-1* mutants (57%; n = 164). However, *gpn-1 (ok377)* mutation further enhanced the defects seen in *lon-2 sdn-1* double mutants from 77% to 84% in triple mutants. Notably, there was a significant increase in the proportion of neurons that failed to migrate at all in *lon-2 sdn-1 gpn-1* triple mutants as compared to *lon-2 sdn-1* double mutants (33% compared 9% of all neurons, respectively; *P*<0.001).

### Role of heparan sulfate biosynthetic enzymes for HSN migration

**Figure 3:**
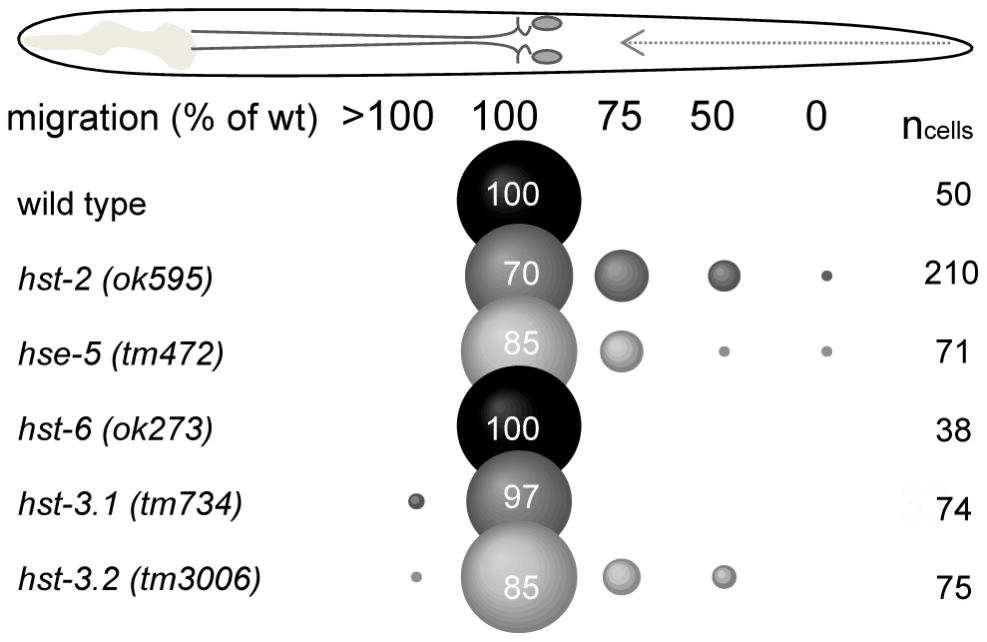
Effects of genetic elimination of HS epimerase or HS O-sulfotransferases on HSN migration. Mutations in *hst-2*, *hse-5* and *hst-3.2*, lead to defects in HSN migration in otherwise wild type genetic background. Mutants in *hst-6* and *hst-3.1* are wild type for HSN migration.

*zh20* is a null allele of *sdn-1*/syndecan [26], whereas *ok449* is an in-frame deletion of the second and third exons [20]. This deletion abolishes two of the three consensus heparan sulfate attachment sites (Ser-Gly) of the SDN-1 protein. Truncated SDN-1 protein is present in *ok449* mutants, but it does not contain any HS chains as detected by monoclonal antibodies against HS (Figure S1 and Methods S1). *zh20* and *ok449* alleles exhibit identical HSN migration defects, suggesting that the role of SDN-1 in HSN migration is predominantly mediated via the HS chains. *rib-1* and *rib-2* are HS polymerases (EXT/EXTL orthologues) required for the biosynthesis of HS chains [23], [24], [31]. Homozygous mutations in *rib-1* (*ok556*) are lethal or sterile [24] and although some homozygous *rib-2* (*tm710*) mutants from heterozygous mothers become adults their progeny dies shortly after gastrulation [31]. Homozygous *rib-2* (*tm710*) mutants from heterozygous mothers were analyzed but were found to have no defects in HSN migration (n_animals_ = 50). The presence of maternal *rib-2* mRNA in the embryo at the time of HSN migration may provide enough RIB-2 protein to mask any defects that result from complete lack of RIB-2. *hst-1* encodes an orthologue of *ndst*, heparan *N*-de-acetylase-*N*-sulfotransferase. Deletion mutation in *ndst*/*hst-1* (*ok1068*) is also homozygous lethal preventing analysis of homozygous offspring from homozygous mutant mothers. Hence the focus on the study was on enzymes modifying HS further in the biosynthetic pathway, mutants of which are all viable. Single mutants in *C. elegans* heparan C5-epimerase, *hse-5* (*tm472)*, and heparan 2-O-sulfotransferases, *hst-2* (*ok595)* have 15% and 30% defects in HSN migration, respectively, whereas single mutants in heparan 6-O-sulfotransferase, *hst-6* (*ok273*), are wild type for HSN migration (Fig. 3 and [19], [21], [26]. *C. elegans* has two orthologues of heparan 3-*O*-sulfotransferases, *hst-3.1* and *hst-3.2*. Single mutants in *hst-3.1* (*tm734*) are wild type for HSN migration, whereas *hst-3.2* (*tm3006*) mutants have 17% defects in HSN migration (Fig. 3).

### Removal of HS 3- and 6-O-sulfation enhance HSN migration defects in HS epimerase mutants

**Figure 4:**
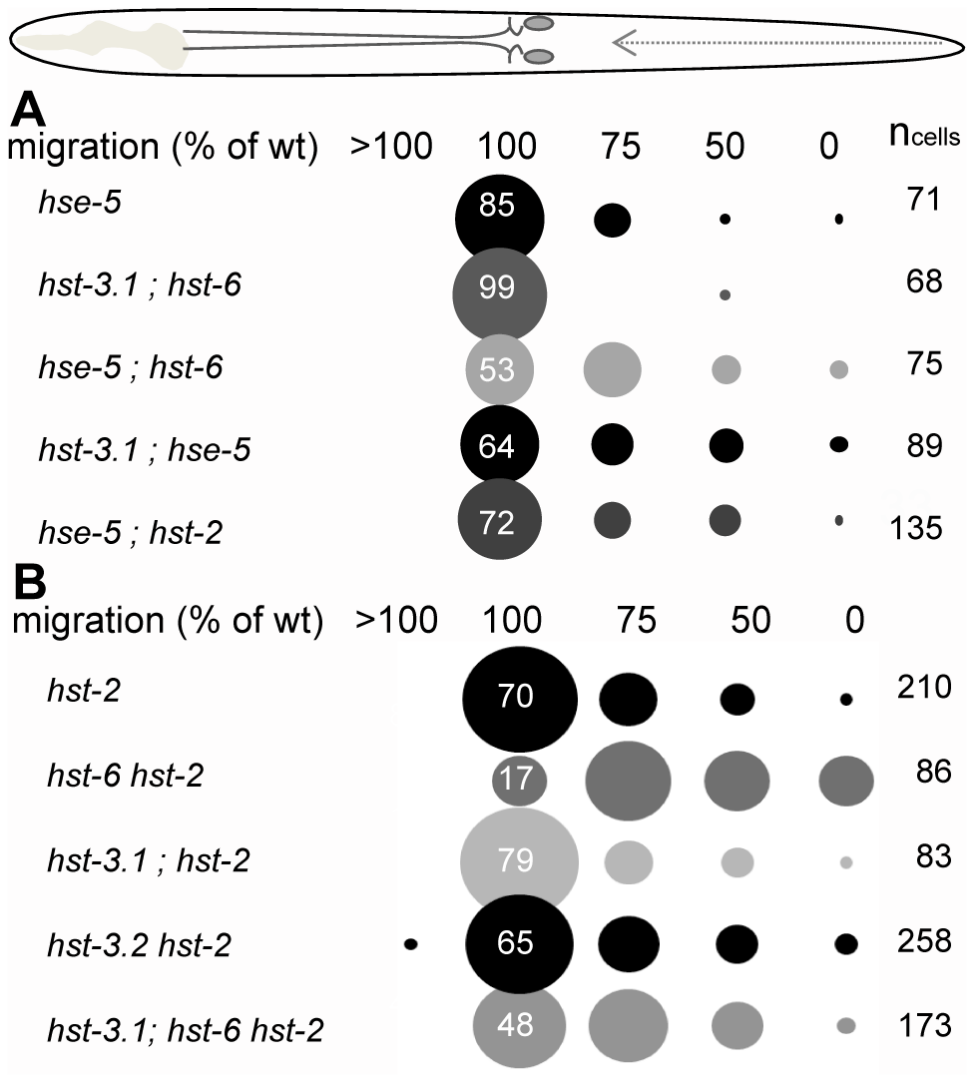
Combinatorial effects of eliminating HS epimerase and O-sulfotransferases for neural migration. (A) Removal of HS 3- and 6-O-sulfotransferases enhances the defects in HS epimerase mutants. *C. elegans* with genetic deletion in either *hst-3.1* or *hst-6* or in combination of both have wild type HSN migration. However, eliminating either *hst-3.1* or *hst-6* in *hse-5* epimerase background significantly enhances the neural phenotype. In contrast, eliminating *hst-2* in *hse-5* background does not alter the combined phenotype as compared to *hst-2* mutants alone. (B) Genetic elimination of HS 2- and 6-O-sulfotransferases has strong additive effects on HSN migration. Note the significant increase in neurons (17%) that fail to migrate at all in *hst-6 hst-2* double mutants. Genetic removal of *hst-3.1* suppresses HSN migration defects of *hst-2* and *hst-6 hst-2* double mutants.

Although eliminating either HST-6 or HST-3.1 alone or in combination as a double mutant of *hst-3.1*; *hst-6* do not affect HSN migration, both *hst-3.1* (*tm734*) and *hst-6* (*ok273*) significantly enhance the defects in *hse-5* epimerase mutants from 15% to 47% and 36% respectively (Fig. 4; *P*<0.001). In contrast, *hse-5 hst-2* double mutants are not more severely affected than *hst-2* single mutants (28% compared to 30% HSN defect; Figs. 3 and 4A).

### Heparan 2-O- and 6-O-sulfotransferase mutants have additive defects in HSN migration

Genetic elimination of multiple O-sulfotransferases has very robust effects on HSN migration in *C. elegans*. Removal of both HS 6- and 2-O-sulfotransferases in *hst-6 hst-2* double mutants leads to 83% (n = 86) HSN migration defect compared to 30% defect in *hst-2* single mutants (Fig. 4B; *P*<0.001). In *hst-2* single mutants only 2% of the HSNs failed to migrate at all and remained positioned in the tail. In contrast, in *hst-6 hst-2* double mutants 17% of all HSNs failed to initiate migration suggesting that removing both HS 2- and 6-O-sulfation severely disrupts molecular interactions required for neuron migration. Intriguingly, further removal of one of the 3-O-sulfotransferases, HST-3.1, from *hst-6 hst-2* double mutants suppresses the HSN migration defect (Fig. 4B). In *hst-3.1; hst-6 hst-2* triple mutants 53% (n = 176) of the HSNs are posteriorly misplaced, with only 2% failing to migrate at all. Removal of HST-3.1 in the *hst-3.1 hst-2* double mutant also weakly suppresses the HSN migration defects of single *hst-2* mutants to 21% (n = 83; *P*<0.05). However, genetic elimination of the other 3-O-sulfotransferase, HST-3.2, from *hst-2* mutant slightly increases the HSN migration defects to 35% (n = 268) in the *hst-3.2 hst-2* double mutant.

### HS modification dependence on HSPG core protein

Specific HS modifications are critical for defined developmental processes in vertebrates. For example, mice lacking heparan 2-O-sulfation have total renal agenesis [11] suggesting that 2-O-sulfates are critical for the growth factors acting in kidney development [32]. HS 2-O-sulfates regulate growth factors involved in the induction and differentiation of kidney mesoteric mesenchyme [33], whereas 6-O-sulfates regulate growth factors required for ureteric bud branching later in kidney development [34]. The HSPGs carrying these HS modifications have not been identified. Neither is it known if the same HSPG undergoes developmental changes in its HS or whether different HSPGs carry the structurally different HS chains. Gene knock-out of a HS biosynthetic enzyme such as HS2ST is anticipated to affect all HSPG core proteins.

**Figure 5:**
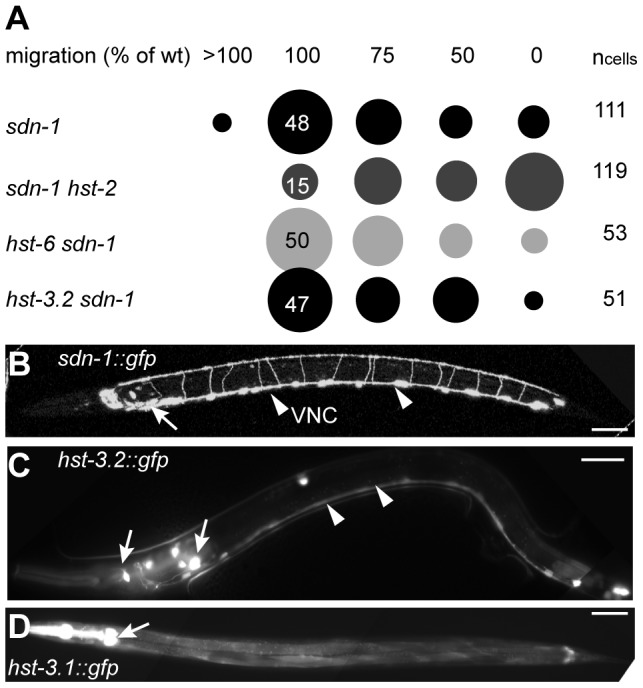
SDN-1 and HS 2-O-sulfate regulate parallel neuron migration pathways. (A) Removing heparan 2-O-sulfation in *sdn-1* mutants significantly enhances HSN migration defects. Note the increased proportion (39%) of HSNs that fail to migrate at all in *sdn-1 hst-2* double mutants. Genetic elimination of *hst-6* or *hst-3.2* in *sdn-1* mutant background does not change the HSN migration phenotype. (B–D) Expression profiles of *sdn-1*, *hst-3.2* and *hst-3.1* reporter genes. (B) *sdn-1* reporter is expressed predominantly in the nervous system, including neurons of the nerve ring (arrow) and in the ventral (VNC; arrowheads) and dorsal nerve cords. (C) *hst-3.2* reporter is also expressed in neurons as seen in some neurons in the nerve ring in the pharynx (arrows) and in the tail ganglia and along ventral nerve cord axon tracks (arrowheads). (D) *hst-3.1* reporter is expressed in the muscle; both the pharyngeal muscle (arrow) and body wall muscle express *hst-3.1* reporter. Scale bars B, D; 50 µm, C; 20 µm.

To gain insight into relationship between HSPG protein cores and specific HS modifications, effects of eliminating HS biosynthetic enzymes in different core protein mutant backgrounds were tested. Eliminating 3- or 6-O-sulfates using *hst-3.2* (*tm3006*) or *hst-6* deletion alleles in *sdn-1* null background did not alter the severity of the HSN phenotype (53% and 52% defect, respectively) as compared to *sdn-1* null mutants alone. In contrast, genetic removal of 2-O-sulfates in the *sdn-1* null background (*sdn-1 hst-2* double mutant) lead to 87% (n = 119) failure of HSN migration to wild type position, with 37% of HSNs failing to migrate at all (Fig. 5). Similarly, double mutants of *hse-5* epimerase and *sdn-1* display more severe defects with 61% of HSNs (n = 81) failing to migrate to wild type position and 33% failing to migrate at all (compared with 17% in *sdn-1* single mutants; *P*<0.01). In contrast, additional removal of 2-O-sulfates from glypican/*lon-2* and collagen XVIII/*cle-1* mutant backgrounds did not significantly alter the neuron defects observed in a single mutant. In *lon-2 hst-2* double mutants 37% (n = 91) and in *cle-1; hst-2* double mutants 24% of all neurons (n = 129) failed to migrate to wild type position. The pattern of migration defects was not significantly changed either.

### Tissue specific expression of HS biosynthetic enzymes and HSPGs

Reporter constructs were used to assess expression patterns of *hst-3.1* and *hst-3.2* and revealed complementary expression patterns for the two 3-O-sulfotransferases. *hst-3.2* reporter is expressed predominantly in neurons (Fig. 5C) whereas *hst-3.1* reporter is expressed predominantly in pharyngeal and body wall muscle (Fig. 5D). Similar complementary expression patterns have previously been shown for the other HS biosynthetic enzymes and HSPGs. *hst-6* is predominantly expressed by neurons, *hst-2* is expressed in the muscle and hypodermis, and *hse-5* is expressed in the hypodermis [19], [21]. *sdn-1* is predominantly expressed in neurons (Fig. 5B; [26], whereas *lon-2* and *cle-1* are expressed in muscle and hypodermis [18], [22].

### Heparan sulfates are required for HSN migration and axon guidance but not process outgrowth

**Figure 6:**
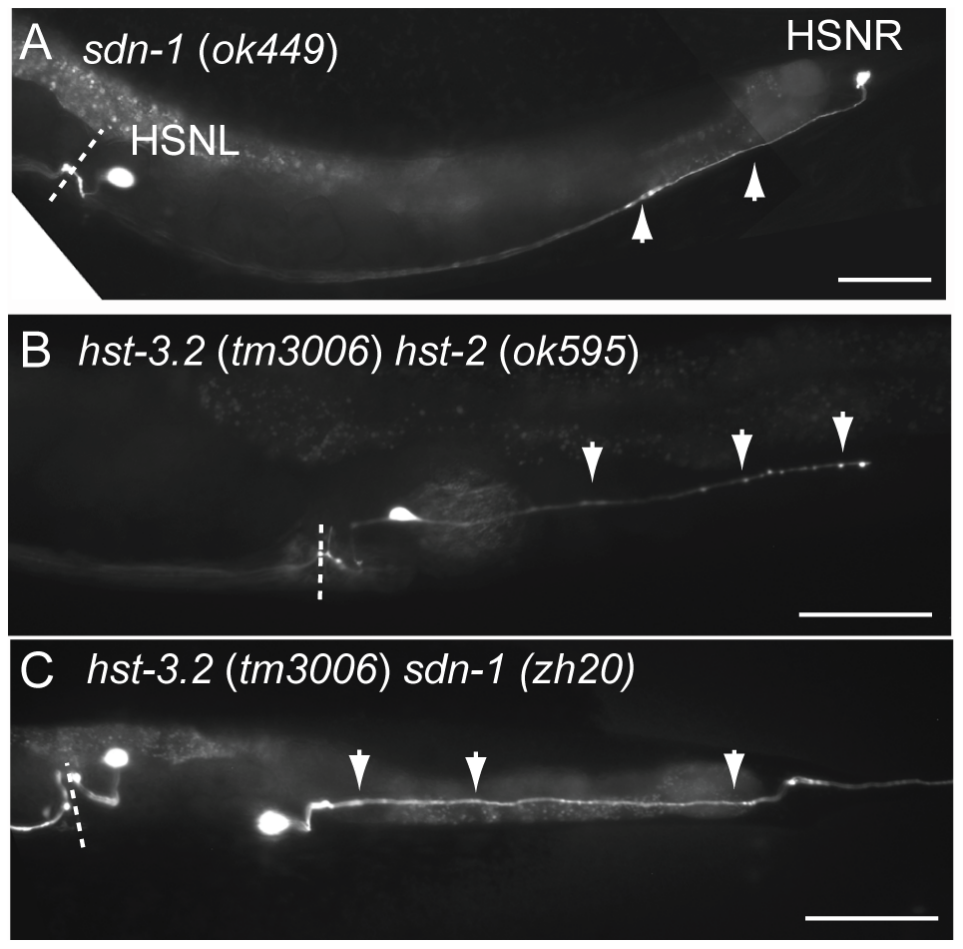
HS and HSPGs are required for axon guidance but not for process outgrowth. All HSNs analysed extended axon projections irrespective of the position of the neural cell body. However, axon guidance was defective in mutants lacking HS biosynthetic enzymes or HSPGs. (A) 17% of all HSNs in *sdn-1* (*ok449*) mutants failed to migrate at all and remain in the tail as the right HSN (HSNR) shown here. The HSN extends axon projection along the ventral nerve cord (arrowheads). In this animal, the left HSN (HSNL) is in wild type position. (B) In some mutants, the HSN cell bodies have migrated to wild type position, but project axons aberrantly. In this *hst-3.2* (*tm3006*) *hst-2* (*ok595*) double mutant, a branch of the HSN axon projects posteriorly (arrowheads). (C) In some mutants, the HSN axons project posteriorly (arrowheads) and completely fail to follow their stereotyped pathway. (A to C); Dashed line indicates the position of vulva. Anterior is to the left, posterior to the right, ventral down in all panels. Scale bars 50 µm.

Deletion mutations in genes encoding for HSPG core proteins and for HS biosynthetic enzyme, led to defects in HSN migration and axon guidance. However, process outgrowth *per se* was not affected in any of the mutants (Fig. 6). In other words, irrespective of whether the HSN cell body had migrated to wild type or aberrant position or failed to migrate at all, the HSNs extended axonal projections, suggesting that the process of axon outgrowth is independent of HS/HSPGs (Fig. 6). The defects in HSN migration and axon guidance were also independent of each other. HSN cell bodies which had failed to migrate at all often extended axons that appeared projecting correctly (Fig. 6A). Conversely, HSN cell bodies which had migrated to correctly to the vulval proximity frequently projected aberrant axons which failed to reach their appropriate targets. HS/HSPGs thus play independent roles in HSN neuron migration and in axon guidance.

## Discussion

Multiple HSPGs were found to play a role in the migration of the HSNs. Glypican/LON-2, collagen XVIII/CLE-1 and syndecan/SDN-1 are required in parallel neuron guidance pathways as genetic elimination of two or all of the HSPGs increases the severity of the HSN migration phenotype. In the absence of all three HSPGs only 20% of HSNs migrate normally. Although mutations in the *gpn-1/*glypican alone do not lead to defects in HSN migration, genetic elimination of *gpn-1* in *lon-2 sdn-1* background significantly increased the proportion of neurons that failed to migrate at all suggesting that in the absence of LON-2, GPN-1 plays a redundant role resulting in increased severity of defects when both glypicans are genetically eliminated.

*sdn-1* is expressed by predominantly in neurons including the HSNs [26], whereas *lon-2* and *cle-1* are expressed in the hypodermis and body wall muscle [18], [22]. This suggests that SDN-1/syndecan is required in the migrating neuron, the HSNs, whereas LON-2/glypican and CLE-1/collagen XVIII are required in the matrix that supports HSN migration and all three HSPGs act in concert. When LON-2/glypican is present, GPN-1/glypican is not required for HSN migration. Moreover, SDN-1 HS chains are important for HSN migration as *sdn-1* null allele and ‘HS-less’ allele display identical phenotypes.

Simultaneous genetic removal of both *hst-2* and *hst-6* results in almost complete loss of HSN migration suggesting that heparan O-sulfation is needed for HSN migration. Biochemical HS disaccharide analysis has shown increased N-sulfation in *hst-2 hst-6* double mutants [35], however, as suggested by this study, glucosamine N-sulfation is not able to functionally compensate for the lack of O-sulfation. Genetic removal of *hst-6* and *hst-3.1* in *hse-5* epimerase mutant background significantly enhances defects in HSN migration whereas removal of *hst-2* in *hse-5* background does not. Biochemical HS disaccharide analysis has shown compensatory 6-O-sulfation in *hse-5; hst-2* double mutants retaining higher total 2- and 6-O-sulfation per 100 disaccharides than that seen in *hse-5; hst-2* double mutants [35]. Heparan 2-O-sulfation occurs predominantly in IdoA residues that have undergone C5-epimerisation (by HSE-5 epimerase) from GluA although some 2-O-sulfation occurs also on GluA residues. It is also plausible that removing both HSE-5 and HST-2 affects the same sugar units in HS, whereas removing HSE-5 and HST-6 or HST-3.1 affects both hexuronic acid and glucosamine units of HS. Hexuronic acids have very flexible conformation, and 2-O-sulfation further increases this flexibility [36]. Changes to this flexibility may affect HS-ligand interactions. In contrast, 3- and 6-O-sulfate groups do not affect conformation of HS to the extent that 2-O-sulfates do [37], which could partly explain why eliminating either HST-3.1 or HST-6, or both, does not affect HSN migration *in vivo* provided other HS modifications are present. Biochemical HS analysis has shown that *hst-6* mutants have increased compensatory 2-O-sulfation, retaining the combined 2- and 6-O-sulfation per 100 disaccharides at wild type levels [35], which may explain why single mutants of *hst-6* display wild type neuron migration phenotype.

3-O-sulfation is the rarest of HS modifications, occurring in less than 5% of HS derived disaccharides. Findings that elimination of *hst-3.1* suppress the HSN migration defects in *hst-2* single and *hst-2 hst-6* double mutant backgrounds suggest that in the absence of 2-O-sulfates or 2- and 6-O-sulfates the presence of 3-O-sulfates is unfavorable for the biological function of the HS. Alternatively, in the absence of 2-O- or 2- and 6-O-sulfation, 3-O-sulfation may be up-regulated. NMR studies have shown that 3-O-sulfates can in some cases influence the conformation of the uronic acid adjacent to the 3-O-sulfated glucosamine [38]. Up-regulation of 3-O-sulfation could thus change the overall HS saccharide conformation and alter ligand binding properties.

Compensatory mechanism in HS biosynthesis has been demonstrated in vertebrates. The classical view of HS biosynthesis is that *N*-deacetylation/*N*-sulfation of glucosamine residues precedes other HS modifications that occur primarily in previously *N*-sulfated domains. However, some 6-O-sulfation is observed in mouse embryonic stem cells deficient of NDST1 and NDST2 and which completely lack *N*-sulfation [39]. The compensatory modifications that occur when one or more HS modification enzymes are eliminated together with the conformational changes to the resulting HS may alter ligand binding properties leading to altered phenotypic outcomes.

*sdn-1 hst-2* double mutants exhibit HSN migration defect that resembles combined elimination of CLE-1, SDN-1 and LON-2 HSPGs. Similarly, genetic elimination of HS 2- and 6-O-sulfation resembles loss of SDN-1 and HST-2, or combined loss of CLE-1, SDN-1 and LON-2 HSPGs. *hst-6 sdn-1* double mutants appear like *sdn-1* single mutants. Genetic elimination of *hst-3.2*, leads to weak defects in HSN migration, but *sdn-1 hst-3.2* double mutants mirror *sdn-1* single mutants. *hst-6* and *hst-3.2* are predominantly expressed by neurons whereas *hst-2* and *hst-3.1* are expressed in the muscle and hypodermis. Combining the normal expression patterns of these genes and the HSN phenotypes observed in the mutants, suggest a model in which SDN-1, which is expressed in the migrating neuron, contains HS chains decorated with 3- and 6-O-sulfates, and the SDN-1 HS chains are important for normal HSN migration. Removal of HST-2 from LON-2 or CLE-1 mutants, did not alter HSN migration defect of the single mutants, suggesting that LON-2/glypican and CLE-1/collagen XVIII, present in the matrix that supports migrating neurons, contain 2-O-sulfated HS required for normal HSN migration. Removal of both HS 2- and 6-O-sulfotransferases resembles loss of all three HSPGs, suggesting that the role of all these HSPGs in neuron migration is predominantly mediated by the HS chains. Based on these findings, it is also plausible that in *C. elegans*, tissue specific expression of different HSPGs and HS O-sulfotransferases in part determines core protein specificity of HS modifications.

In conclusion, a neural development model in *C. elegans* was used to address relationship between different HSPG core proteins and distinct HS modifications for biological function *in vivo*. Genetic analysis demonstrated that SDN-1/syndecan, LON-2/glypican and CLE-1/collagen XVIII are required in parallel guidance pathways and genetic elimination of all three HSPGs results in almost complete failure of neural migration. The different HSPG core proteins require distinct HS modifications to mediate *in vivo* functions. The core protein is thus not a carrier of HS chains with any or random modifications. In *C. elegans* this core protein-HS specificity may in part be imposed by tissue specific expression of the genes encoding for HSPGs and HS biosynthetic enzymes. This is the first demonstration of relationship between HS modifications and distinct HSPG core proteins in a defined biological process and provides a novel insight into the biological functions of HS and HSPGs. Proteoglycan core protein specificity should be considered when interpreting data or considering therapies relating to distinct HS structures.

## Supporting Information

Figure S1 **Biochemical analysis of** ***sdn-1*** **mutants.** SDN-1 is absent in both *zh20* (null allele) and *ok449* (in-frame deletion abolishing HS attachment sites) mutants as detected by monoclonal antibodies recognising the HS “stub” as a result of treatment with heparinase III. Proteins were purified using anion-exchange chromatography (DEAE), which enriches for negatively charged HSPGs. SDN-1 core protein is however present in *ok449* mutants as detected by anti-SDN-1 antibodies following immunoprecipitation using anti-SDN-1 antibodies.
(TIF)

Methods S1 **HSPG purification and Western blotting.** Mixed stage *C. elegans* were lysed and proteins purified essentially as described [40] with the following modifications. Total protein lysates were either passed through DEAE anion-exchange matrix (GE Healthcare Life Sciences) and after washing the matrix with 0.25 M NaCl, bound proteins were eluted with 1.5 M NaCl. Alternatively, SDN-1 was immuno-precipitated with rabbit polyclonal anti-SDN-1 antibodies made against synthetic peptide corresponding to the entire cytoplasmic domain of SDN-1. DEAE purified proteins were treated with heparitinase III (Ibex, Canada) as described [27]. Proteins were separated on 10% SDS-PAGE and blotted to Immobilon-P membranes (Millipore, USA). Heparitinase III treated samples were detected with monoclonal anti-HS stub antibody 3G10 (Seikagaku, Japan) followed by anti-mouse HRP secondary antibody (GE Healthcare) and immunoprecipitated samples were detected with anti-SDN-1 antibody followed by anti-rabbit HRP conjugated secondary antibody (GE Healthcare). Western blots were visualised using ECL Chemilumenescence detection kit (Biological Industries, Beit Haemek, Israel).
(PDF)
