# Supplementary figures and images for: Combinatorial Roles of Heparan Sulfate Proteoglycans and Heparan Sulfates in Caenorhabditis elegans Neural Development
Source: PLoS One. 2014 Jul 23;9(7):e102919. doi: 10.1371/journal.pone.0102919 (PMC4108370; doi:10.1371/journal.pone.0102919)

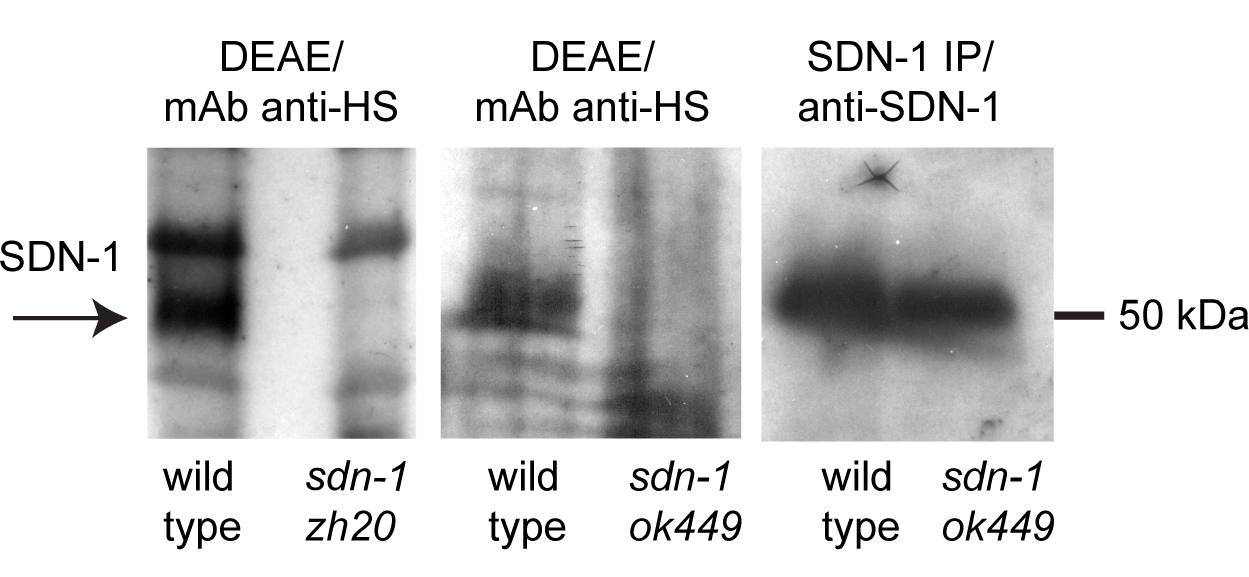

Supplement: Figure S1 — Biochemical analysis of sdn-1 mutants. SDN-1 is absent in both zh20 (null allele) and ok449 (in-frame deletion abolishing HS attachment sites) mutants as detected by monoclonal antibodies recognising the HS “stub” as a result of treatment with heparinase III. Proteins were purified using anion-exchange chromatography (DEAE), which enriches for negatively charged HSPGs. SDN-1 core protein is however present in ok449 mutants as detected by anti-SDN-1 antibodies following immunoprecipitation using anti-SDN-1 antibodies. (TIF) [file pone.0102919.s001.tif]
