# Supplementary material for: Combinatorial Roles of Heparan Sulfate Proteoglycans and Heparan Sulfates in Caenorhabditis elegans Neural Development
Source: PLoS One. 2014 Jul 23;9(7):e102919. doi: 10.1371/journal.pone.0102919 (PMC4108370; doi:10.1371/journal.pone.0102919)
Supplement: Methods S1 — HSPG purification and Western blotting. Mixed stage C. elegans were lysed and proteins purified essentially as described [40] with the following modifications. Total protein lysates were either passed through DEAE anion-exchange matrix (GE Healthcare Life Sciences) and after washing the matrix with 0.25 M NaCl, bound proteins were eluted with 1.5 M NaCl. Alternatively, SDN-1 was immuno-precipitated with rabbit polyclonal anti-SDN-1 antibodies made against synthetic peptide corresponding to the entire cytoplasmic domain of SDN-1. DEAE purified proteins were treated with heparitinase III (Ibex, Canada) as described [27]. Proteins were separated on 10% SDS-PAGE and blotted to Immobilon-P membranes (Millipore, USA). Heparitinase III treated samples were detected with monoclonal anti-HS stub antibody 3G10 (Seikagaku, Japan) followed by anti-mouse HRP secondary antibody (GE Healthcare) and immunoprecipitated samples were detected with anti-SDN-1 antibody followed by anti-rabbit HRP conjugated secondary antibody (GE Healthcare). Western blots were visualised using ECL Chemilumenescence detection kit (Biological Industries, Beit Haemek, Israel). (PDF) [file pone.0102919.s002.pdf]

Supporting text

Supporting methods

HSPG purification and Western blotting - Mixed stage *C. elegans* were lysed and proteins purified essentially as described (1) with the following modifications. Total protein lysates were either passed through DEAE anion-exchange matrix (GE Healthcare Life Sciences) and after washing the matrix with 0.25 M NaCl, bound proteins were eluted with 1.5 M NaCl. Alternatively, SDN-1 was immune-precipitated with rabbit polyclonal anti-SDN-1 antibodies made against synthetic peptide corresponding to the entire cytoplasmic domain of SDN-1. DEAE purified proteins were treated with heparitinase III (Ibex, Canada) as described (2). Proteins were separated on 10 % SDS-PAGE and blotted to Immobilon-P membranes (Millipore, USA). Heparitinase III treated samples were detected with monoclonal anti-HS stub antibody 3G10 (Seikagaku, Japan) followed by anti-mouse HRP secondary antibody (GE Healthcare) and immunoprecipitated samples were detected with anti-SDN-1 antibody followed by anti-rabbit HRP conjugated secondary antibody (GE Healthcare). Western blots were visualised using ECL Chemilumenescence detection kit (Biological Industries, Beit Haemek, Israel).

Supporting figure legends

Supporting figure 1. Biochemical analysis of *sdn-1* mutants. SDN-1 is absent in both *zh20* (null allele) and *ok449* (in-frame deletion abolishing HS attachment sites) mutants as detected by monoclonal antibodies recognising the HS "stub" as a result of treatment with heparinase III. Proteins were purified using anion-exchange chromatography (DEAE), which enriches for negatively charged HSPGs. SDN-1 core protein is however present in *ok449* mutants as detected by anti-SDN-1 antibodies following immunoprecipitation using anti-SDN-1 antibodies.

Supporting References

1. Polanska UM, Duchesne L, Harries JC, Fernig DG, & Kinnunen TK (2009) N-Glycosylation regulates fibroblast growth factor receptor/EGL-15 activity in Caenorhabditis elegans in vivo. *J Biol Chem* 284(48):33030-33039.
2. Hudson ML, Kinnunen T, Cinar HN, & Chisholm AD (2006) C. elegans Kallmann syndrome protein KAL-1 interacts with syndecan and glypican to regulate neuronal cell migrations. *Dev Biol* 294(2):352-365.
